# Comparison of the role of alcohol consumption and qualitative abdominal fat on NAFLD and MAFLD in males and females

**DOI:** 10.1038/s41598-022-20124-8

**Published:** 2022-09-26

**Authors:** Masahiro Sogabe, Toshiya Okahisa, Takeshi Kurihara, Miwako Kagawa, Hiroyuki Ueda, Tomoyuki Kawaguchi, Akira Fukuya, Kaizo Kagemoto, Hironori Tanaka, Yoshifumi Kida, Tetsu Tomonari, Tatsuya Taniguchi, Koichi Okamoto, Hiroshi Miyamoto, Yasushi Sato, Masahiko Nakasono, Tetsuji Takayama

**Affiliations:** 1 Department of Gastroenterology and Oncology, Tokushima University Graduate School of Biomedical Sciences, 3-18-15 Kuramoto-cho, Tokushima, 770-8503 Japan; 2 Health Service, Counseling and Accessibility Center, Tokushima University, Tokushima, Japan; 3 Department of Internal Medicine, Shikoku Central Hospital of the Mutual Aid Association of Public School Teachers, Shikokuchuo, Japan; 4 Department of Internal Medicine, Tsurugi Municipal Handa Hospital, Tsurugi, Japan

**Keywords:** Non-alcoholic fatty liver disease, Metabolic disorders

## Abstract

The clinical difference between nonalcoholic fatty liver disease (NAFLD) and metabolic-associated fatty liver disease (MAFLD) between the two sexes is unclear. This study aimed to determine the influences of alcohol consumption and qualitative abdominal fat between male and female patients with NAFLD and MAFLD. This cross-sectional study examined 11,766 participants who underwent health check-ups comparing lifestyle habits, biochemical features, and noninvasive liver fibrosis scores, between non-MAFLD and MAFLD groups. Furthermore, differences in alcohol consumption and qualitative abdominal fat were examined between male and female patients with NAFLD and MAFLD. The prevalence of metabolic dysregulation, ratio of visceral fat area to subcutaneous fat area, and noninvasive liver fibrosis scores were significantly higher in male patients with MAFLD than in those with NAFLD (*p* < 0.05), but these were not significantly different in female patients. Among male patients with an alcohol consumption of > 70 g/week, several noninvasive liver fibrosis scores were significantly higher in the MAFLD group than in the NAFLD group (all *p* < 0.05). The influences of alcohol consumption and qualitative abdominal fat on NAFLD and MAFLD were different between sexes. The development of liver fibrosis should be considered in male patients with MAFLD who exceed mild drinking.

## Introduction

Nonalcoholic fatty liver disease (NAFLD) is currently the most common liver disease in Asian and Western countries, and it may lead to nonalcoholic steatohepatitis, cirrhosis, liver failure, and hepatocellular carcinoma^1–5^. NAFLD is diagnosed by the presence of hepatic steatosis in the absence of excessive alcohol consumption or other liver diseases, and it is known to be strongly associated with metabolic syndrome^6,7^. However, the presence of metabolic dysregulation has not been used in the definition of NAFLD. Recently, metabolic-associated fatty liver disease (MAFLD) was proposed from an international expert consensus in 2020^8^, which highlights the association between fatty liver disease and metabolic dysregulation and does not require the exclusion of excessive alcohol consumption, viral hepatitis, or other liver diseases^8,9^. Although there are many reports on the influences of alcohol consumption and abdominal fat on NAFLD^10–14^, the utility of MAFLD in clinical practice and the influences of alcohol consumption, qualitative abdominal fat, and sex when distinguishing between MAFLD and NAFLD are not sufficiently clear because the criteria for MAFLD are new and do not assess alcohol consumption and qualitative abdominal fat. Therefore, this study aimed to investigate the clinical factors associated with MAFLD (including MAFLD subgroups) to clarify the clinical differences between NAFLD and MAFLD based on alcohol consumption, qualitative abdominal fat, and sex.

## Results

### Baseline characteristics of non-MAFLD and MAFLD patients

**Figure 1:**
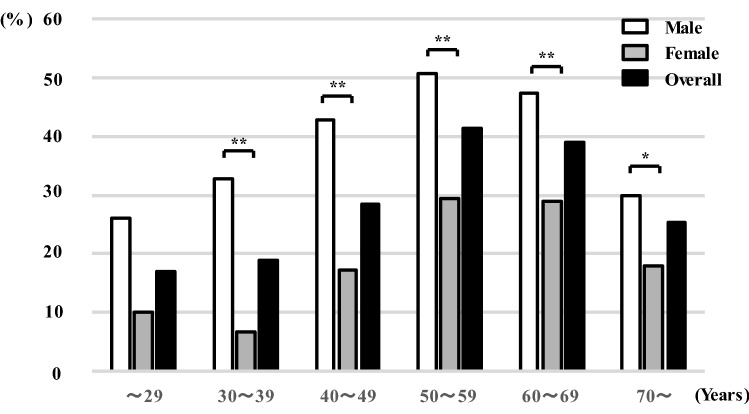
Comparison of the prevalence of MAFLD between sex and age groups. The white bar indicates male patients. The gray bar indicates female patients. The black bar indicates overall patients. *p < 0.05, **p < 0.001. MAFLD, metabolic-associated fatty liver disease.

Among 11,766 participants, the prevalence of MAFLD in male and female patients was 46.6% and 23.5%, respectively (Table 1). The aspartate aminotransferase (AST)-to-platelet ratio index (APRI) and NAFLD fibrosis score (NFS) were significantly higher in patients with MAFLD than in those with non-MAFLD (both *p* < 0.001); however, the AST/ alanine aminotransferase (ALT) ratio (AAR) and Fibrosis-4 (FIB-4) Index were significantly lower in patients with MAFLD than in those with non-MAFLD (both *p* < 0.001). The prevalence of MAFLD in male participants increased in their 50 s and decreased thereafter; in female participants, the prevalence of MAFLD decreased in their 30 s, increased until their 50 s, and decreased from their 60 s (Fig. 1). The prevalence of MAFLD differed significantly between male and female patients after their 20 s (all *p* < 0.05).

**Table 1:** Baseline characteristics of the non-MAFLD and MAFLD groups.

		Total patients	Male (n = 6,106)			Female (n = 5,660)		
			Non-MAFLD	MAFLD	*p*-value	Non-MAFLD	MAFLD	*p*-value
Number		11,766	3,261	2,845		4,328	1,332	
Age	(years)	52.3 ± 8.9	52.6 ± 9.5	53.5 ± 7.7	< 0.001	50.7 ± 9.4	54.1 ± 7.0	< 0.001
BMI	(kg/m^2^)	23.7 ± 3.7	22.9 ± 2.5	26.4 ± 3.3	< 0.001	21.5 ± 2.8	26.7 ± 4.1	< 0.001
WC	(cm)	83.7 ± 10.0	81.9 ± 7.1	91.4 ± 8.5	< 0.001	77.7 ± 8.0	91.1 ± 9.3	< 0.001
Current smoking		1,421 (12.1)	686 (21.0)	645 (22.7)	0.128	74 (1.7)	16 (1.2)	0.212
Drinking		6,607 (56.2)	2,359 (72.3)	1,972 (69.3)	< 0.05	1,803 (41.7)	473 (35.5)	< 0.001
**Alcohol consumption (g/week)**								
	None		902 (27.7)	873 (30.7)	< 0.05	2,525 (58.3)	859 (64.5)	< 0.01
	0.1–69.9		845 (25.9)	665 (23.4)		1,256 (29.0)	317 (23.8)	
	70–139.9		1,068 (32.8)	885 (31.1)		450 (10.4)	130 (9.8)	
	140–279.9		362 (11.1)	353 (12.4)		78 (1.8)	19 (1.4)	
	≥ 280		84 (2.6)	69 (2.4)		19 (0.4)	7 (0.5)	
Regular exercise		3,074 (26.1)	1,268 (38.9)	838 (29.5)	< 0.001	770 (17.8)	198 (14.9)	< 0.05
Eating before going to bed		4,633 (39.4)	1,329 (40.8)	1,215 (42.7)	0.125	1,556 (36.0)	533 (40.0)	< 0.01
Eating breakfast		1,186 (10.1)	403 (12.4)	344 (12.1)	0.754	345 (8.0)	94 (7.1)	0.292
SBP	(mmHg)	123.5 ± 17.2	123.7 ± 16.2	131.0 ± 16.0	< 0.001	116.5 ± 15.8	130.0 ± 16.8	< 0.001
DBP	(mmHg)	79.3 ± 12.6	80.5 ± 11.9	86.2 ± 12.0	< 0.001	73.2 ± 11.0	81.6 ± 11.2	< 0.001
Hypertension		5,394 (45.8)	1,532 (47.0)	1,990 (69.9)	< 0.001	1,083 (25.0)	789 (59.2)	< 0.001
T-CHO	(mg/dL)	212.0 ± 34.9	207.0 ± 32.7	211.0 ± 35.5	< 0.001	212.8 ± 35.7	223.7 ± 33.4	< 0.001
TG	(mg/dL)	109.8 ± 81.7	107.5 ± 69.5	159.0 ± 116.9	< 0.001	76.0 ± 38.6	120.7 ± 64.0	< 0.001
HDL-C	(mg/dL)	66.9 ± 17.6	65.1 ± 16.0	54.8 ± 12.6	< 0.001	77.2 ± 16.4	64.1 ± 14.7	< 0.001
LDL-C	(mg/dL)	129.2 ± 31.0	126.4 ± 29.4	133.4 ± 31.9	< 0.001	124.9 ± 30.5	141.4 ± 29.8	< 0.001
Dyslipidemia		3,307 (28.1)	751 (23.0)	1,489 (52.3)	< 0.001	501 (11.6)	566 (42.5)	< 0.001
FPG	(mg/dL)	99.8 ± 17.5	99.2 ± 14.7	108.9 ± 23.6	< 0.001	93.1 ± 9.6	103.8 ± 18.3	< 0.001
HbA1c	(%)	5.6 ± 0.57	5.5 ± 0.43	5.9 ± 0.84	< 0.001	5.5 ± 0.30	5.8 ± 0.59	< 0.001
IGT		5,891 (50.1)	1,482 (45.4)	2,051 (72.1)	< 0.001	1,447 (33.4)	911 (68.4)	< 0.001
UA	(mg/dL)	5.3 ± 1.4	5.8 ± 1.2	6.3 ± 1.3	< 0.001	4.3 ± 0.9	5.1 ± 1.1	< 0.001
ALT	(IU/L)	23.8 ± 17.2	22.4 ± 11.9	35.4 ± 22.0	< 0.001	16.4 ± 11.5	26.3 ± 18.1	< 0.001
AST	(IU/L)	24.3 ± 10.8	24.4 ± 9.7	28.5 ± 12.3	< 0.001	21.4 ± 9.6	24.7 ± 10.4	< 0.001
GGT	(IU/L)	37.3 ± 45.7	41.5 ± 48.9	58.8 ± 62.0	< 0.001	20.9 ± 18.9	34.4 ± 36.1	< 0.001
AAR		1.18 ± 0.40	1.18 ± 0.38	0.90 ± 0.31	< 0.001	1.40 ± 0.35	1.07 ± 0.34	< 0.001
APRI		0.27 ± 0.20	0.28 ± 0.22	0.32 ± 0.22	< 0.001	0.23 ± 0.17	0.25 ± 0.14	< 0.001
FIB-4 index		1.21 ± 0.60	1.30 ± 0.71	1.19 ± 0.58	< 0.001	1.19 ± 0.56	1.09 ± 0.45	< 0.001
NFS		− 1.89 ± 1.13	− 1.88 ± 1.17	− 1.62 ± 1.09	< 0.001	− 2.14 ± 1.10	− 1.70 ± 1.08	< 0.001

Data are presented as the mean ± standard deviation or number (%) for categorical variables. *p*-values are based on the χ^2^-test or Mann–Whitney U-test. *p*-values of three or more groups were determined using the m × n χ^2^ test.

*AAR* AST/ALT ratio; *ALT* alanine aminotransferase; *APRI* AST-to-platelet ratio index; *AST* aspartate aminotransferase; *BMI* body mass index; *DBP* diastolic blood pressure; *FIB-4* Fibrosis-4; *FPG* fasting plasma glucose; *GGT* gamma-glutamyl transpeptidase; *HbA1c* hemoglobin A1c; *HDL-C* high-density lipoprotein cholesterol; *IGT* impaired glucose tolerance; *LDL-C* low-density lipoprotein cholesterol; *MAFLD* metabolic-associated fatty liver disease; *NFS* nonalcoholic fatty liver disease (NAFLD) fibrosis score; *SBP* systolic blood pressure; *T-CHO* total cholesterol; *TG* triglyceride; *UA* uric acid; *WC* waist circumference.

*p* < 0.05 was considered statistically significant.

### Comparison of baseline characteristics between MAFLD subgroups

There was a significant difference in the quantity of alcohol consumption between the MAFLD subgroups (χ^2^(8, N = 4,177) = 24.5, *p* < 0.005) (Table 2). The prevalence of metabolic dysregulation (e.g., hypertension, dyslipidemia, and Impaired glucose intolerance [IGT]) and the level of liver enzymes (e.g., ALT, AST, and gamma-glutamyl transpeptidase [GGT]) were the highest in Group 3 and the lowest in Group 1 (all *p* < 0.001). The APRI and NFS were also the highest in Group 3 and the lowest in Group 1 (both *p* < 0.001); however, the AAR was the highest in Group 1 and lowest in Group 3 (*p* < 0.001).

**Table 2:** Comparison of baseline characteristics between the three MAFLD subgroups.

		Group 1	Group 2	Group 3	*p*-value
Number		609	2,968	600	
Sex	(male)	387 (63.5)	2,007 (67.6)	451 (75.2)	< 0.001
Age	(years)	54.3 ± 7.9	53.2 ± 7.5	55.7 ± 6.6	< 0.001
BMI	(kg/m^2^)	22.2 ± 1.5	27.0 ± 3.2	28.3 ± 3.8	< 0.001
WC	(cm)	81.9 ± 4.3	92.4 ± 8.0	95.6 ± 9.2	< 0.001
Current smoking		100 (16.4)	446 (15.0)	115 (19.2)	< 0.05
Drinking		356 (58.5)	1,760 (59.3)	329 (54.8)	0.129
**Alcohol consumption (g/week)**					
	None	253 (41.5)	1,208 (40.7)	271 (45.2)	< 0.005
	0.1–69.9	151 (24.8)	700 (23.6)	131 (21.8)	
	70–139.9	150 (24.6)	750 (25.3)	115 (19.2)	
	140–279.9	52 (8.5)	257 (8.7)	63 (10.5)	
	≥ 280	3 (0.5)	53 (1.8)	20 (3.3)	
Regular exercise		140 (23.0)	734 (24.7)	162 (27.0)	0.268
Eating before going to bed		221 (36.3)	1,281 (43.2)	246 (41.0)	< 0.01
Eating breakfast		63 (10.3)	323 (10.9)	52 (8.7)	0.269
SBP	(mmHg)	123.8 ± 14.8	131.0 ± 15.8	135.8 ± 17.6	< 0.001
DBP	(mmHg)	79.6 ± 10.7	85.3 ± 11.9	86.8 ± 12.1	< 0.001
Hypertension		284 (46.6)	1,983 (66.8)	512 (85.3)	< 0.001
Medication for hypertension		94 (15.4)	691 (23.3)	287 (47.8)	< 0.001
T-CHO	(mg/dL)	215.0 ± 37.0	216.2 ± 33.8	209.3 ± 40.5	< 0.001
TG	(mg/dL)	127.9 ± 78.5	145.0 ± 97.1	174.5 ± 148.9	< 0.001
HDL-C	(mg/dL)	62.4 ± 15.4	57.7 ± 13.7	53.6 ± 12.8	< 0.001
LDL-C	(mg/dL)	133.9 ± 33.9	137.4 ± 30.2	130.7 ± 34.4	< 0.001
Dyslipidemia		209 (34.3)	1,408 (47.4)	438 (73.0)	< 0.001
Medication for dyslipidemia		73 (12.0)	478 (16.1)	247 (41.2)	< 0.001
FPG	(mg/dL)	98.7 ± 9.9	101.8 ± 11.0	143.4 ± 34.5	< 0.001
HbA1c	(%)	5.6 ± 0.3	5.7 ± 0.4	7.1 ± 1.2	< 0.001
IGT		258 (42.4)	1,665 (56.1)	598 (99.7)	< 0.001
Medication for DM		3 (0.5)	39 (1.3)	354 (59.0)	< 0.001
UA	(mg/dL)	5.6 ± 1.3	6.0 ± 1.3	5.6 ± 1.2	< 0.001
ALT	(IU/L)	24.8 ± 14.5	32.9 ± 20.9	38.5 ± 25.9	< 0.001
AST	(IU/L)	24.4 ± 10.0	27.3 ± 11.0	30.3 ± 16.0	< 0.001
GGT	(IU/L)	43.2 ± 45.7	51.3 ± 57.0	57.2 ± 61.2	< 0.001
AAR		1.09 ± 0.35	0.95 ± 0.32	0.87 ± 0.28	< 0.001
APRI		0.26 ± 0.16	0.29 ± 0.19	0.35 ± 0.27	< 0.001
FIB-4 index		1.20 ± 0.55	1.13 ± 0.50	1.27 ± 0.70	< 0.001
NFS		− 2.09 ± 1.09	− 1.67 ± 1.06	− 1.06 ± 0.95	< 0.001

Data are presented as the mean ± standard deviation or number (%) for categorical variables.

*p*-values are based on the m × n χ^2^-test or Kruskal–Wallis test.

*AAR* AST/ALT ratio; *ALT* alanine aminotransferase; *APRI* AST-to-platelet ratio index; *AST* aspartate aminotransferase; *BMI* body mass index; *DBP* diastolic blood pressure; *DM* diabetes mellitus; *FIB-4* Fibrosis-4; *FPG* fasting plasma glucose; *GGT* gamma-glutamyl transpeptidase; *HbA1c* hemoglobin A1c; *HDL-C* high-density lipoprotein cholesterol; *IGT* impaired glucose tolerance; *LDL-C* low-density lipoprotein cholesterol; *MAFLD* metabolic-associated fatty liver disease; *NFS* nonalcoholic fatty liver disease (NAFLD) fibrosis score; *SBP* systolic blood pressure; *T-CHO* total cholesterol; *TG* triglyceride; *UA* uric acid; *WC* waist circumference.

*p* < 0.05 was considered statistically significant.

### Association between MAFLD and lifestyle habits, metabolic dysregulation, liver enzymes, and noninvasive liver fibrosis scores

Among male patients, the odds ratio (OR) (95% confidence interval [CI]) of drinking for MAFLD was 0.830 (0.773–0.965, *p* < 0.01). In analysis of the quantity of alcohol consumption, the OR of drinking with 70–139.9 g/week and 140–279.9 g/week was 0.896 (0.816–0.983, *p* < 0.05) and 0.931 (0.885–0.980, *p* < 0.01), respectively and the OR in all drinking categories was < 1, regardless of quantity. Among female patients, the OR for MAFLD in drinking was 0.853 (0.724–1.006, *p* = 0.058) and the OR of drinking with ≥ 280 g/week was 2.092 (0.464–9.430, *p* = 0.337) (Table 3). The OR of regular exercise in male and female patients was 0.726 (0.637–0.826, *p* < 0.001) and 0.712 (0.571–0.888, *p* < 0.005), respectively. The ORs associated with metabolic dysregulation and elevation of liver enzymes were > 1 (all, *p* < 0.001) in both sexes. The OR of elevated APRI and NFS in male and female patients was 2.106 (1.708–2.596, *p* < 0.001), 1.742 (1.568–1.935, *p* < 0.001) and 2.616 (1.845–3.710, *p* < 0.001), 2.237 (1.964–2.548, *p* < 0.001), respectively; however, the ORs of elevated AAR and FIB-4 index were < 1 (all, *p* < 0.001), regardless of sex.

**Table 3:** Odds ratios for MAFLD in each category of lifestyle habits, metabolic dysregulation, liver enzymes, and noninvasive liver fibrosis scores.

	Male (n = 6,106)		Female (n = 5,660)	
	OR (95% CI)	aOR (95% CI)	OR (95% CI)	aOR (95% CI)
**Lifestyle habits**				
Current smoking	1.101 (0.975–1.243)	1.156 (0.998–1.338)	0.699 (0.406–1.204)	0.867 (0.451–1.666)
Drinking	0.864 (0.773–0.965)	0.830 (0.773–0.965)	0.771 (0.679–0.876)	0.853 (0.724–1.006)
**Alcohol consumption (g/week)**				
None	1	1	1	1
0.1–69.9	0.875 (0.753–1.016)	0.869 (0.724–1.044)	0.800 (0.689–0.931)	0.909 (0.751–1.100)
70–139.9	0.938 (0.869–1.012)	0.896 (0.816–0.983)	0.679 (0.541–0.853)	0.647 (0.484–0.864)
140–279.9	0.947 (0.907–0.988)	0.931 (0.885–0.980)	0.786 (0.600–1.029)	0.996 (0.702–1.414)
≥ 280	0.967 (0.872–1.072)	0.945 (0.838–1.066)	1.470 (0.441–4.893)	2.092 (0.464–9.430)
Regular exercise	0.656 (0.590–0.730)	0.726 (0.637–0.826)	0.807 (0.681–0.956)	0.712 (0.571–0.888)
Eating before going to bed	1.084 (0.979–1.200)	1.021 (0.900–1.158)	1.188 (1.048–1.348)	1.006 (0.851–1.189)
Eating breakfast	0.975 (0.837–1.137)	1.070 (0.885–1.293)	0.877 (0.692–1.111)	0.849 (0.621–1.160)
**Metabolic dysregulation**				
Hypertension	2.627 (2.364–2.919)	1.377 (1.210–1.567)	4.354 (3.826–4.954)	1.813 (1.531–2.146)
Dyslipidemia	3.670 (3.288–4.096)	2.281 (2.007–2.593)	5.644 (4.892–6.513)	2.924 (2.442–3.501)
IGT	3.101 (2.786–3.451)	2.166 (1.906–2.462)	4.308 (3.777–4.915)	2.331 (1.969–2.759)
**Liver enzymes**				
ALT (≥ 31 IU/L)	5.034 (4.457–5.686)	3.184 (2.758–3.675)	7.234 (5.943–8.805)	3.446 (2.658–4.468)
AST (≥ 31 IU/L)	2.682 (2.352–3.058)	1.886 (1.607–2.214)	3.672 (3.019–4.467)	2.147 (1.635–2.820)
GGT (≥ 51 IU/L)	2.503 (2.231–2.807)	1.979 (1.727–2.269)	3.292 (2.674–4.052)	1.876 (1.430–2.461)
**Noninvasive liver fibrosis scores**				
AAR (≥ 1)	0.221 (0.198–0.246)		0.130 (0.113–0.150)	
APRI (≥ 0.5)	2.106 (1.708–2.596)		2.616 (1.845–3.710)	
FIB-4 index (age < 65: ≥ 1.3; age ≥ 65: ≥ 2.0)				
	0.745 (0.669–0.830)		0.682 (0.591–0.788)	
NFS (age < 65: ≥ − 1.455; age ≥ 65: ≥ 0.12)				
	1.742 (1.568–1.935)		2.237 (1.964–2.548)	

Factors with significant influence on the prevalence of MAFLD were determined using multivariate logistic regression analysis. The aOR is the adjusted OR for age, BMI, and WC.

*AAR* AST/ALT ratio; *ALT* alanine aminotransferase; *APRI* AST-to-platelet ratio index; *AST* aspartate aminotransferase; *CI* confidence interval; *FIB-4* Fibrosis-4; *GGT* gamma-glutamyl transpeptidase; *IGT* impaired glucose tolerance; *MAFLD* metabolic-associated fatty liver disease; *NFS* nonalcoholic fatty liver disease (NAFLD) fibrosis score; *OR* odds ratio.

### Comparison of clinical characteristics between NAFLD and MAFLD patients

The prevalence of NAFLD and MAFLD was 32.7% and 46.6% in male patients and 22.2% and 23.5% in female patients, respectively (Table 4). In participants with a fatty liver (n = 4,247), the prevalence of overlapping NAFLD and MAFLD was 75.4% (3,203/4,247) and the prevalence of only NAFLD or MAFLD was 24.2% (1,026/4,247) (Supplementary Figure S1). There was a significant difference in the quantity of alcohol consumption between NAFLD and MAFLD in male and female patients (χ^2^(5, N = 4,841) = 410.1, *p* < 0.001) and (χ^2^(5, N = 2,591) = 26.1, *p* < 0.001), respectively). The values of several clinical factors were significantly higher in male patients with MAFLD than in those with NAFLD; however, there were no significant differences in characteristics between female patients with MAFLD and those with NAFLD, except drinking and the quantity of alcohol consumption. Four noninvasive liver fibrosis scores were significantly higher in male patients with MAFLD than in male patients with NAFLD (all *p* < 0.05). Additionally, we compared female patients aged < 50 years with female patients aged ≥ 50 years to evaluate the influence of menopause (Supplementary Table S1) and found that the results were comparable between these two age groups.

**Table 4:** Comparison of characteristics between NAFLD and MAFLD.

		Male			Female		
		NAFLD	MAFLD	*p*-value	NAFLD	MAFLD	*p*-value
Number		1,996	2,845		1,259	1,332	
Age	(years)	52.8 ± 8.1	53.5 ± 7.7	< 0.005	54.0 ± 7.0	54.1 ± 7.0	0.745
BMI	(kg/m^2^)	26.5 ± 3.5	26.4 ± 3.3	0.139	26.6 ± 4.1	26.7 ± 4.1	0.537
WC	(cm)	91.7 ± 8.9	91.4 ± 8.5	0.675	90.7 ± 9.4	91.1 ± 9.3	0.319
Current smoking		419 (21.0)	645 (22.7)	0.169	12 (0.9)	16 (1.2)	0.573
Drinking		1,119 (56.1)	1,972 (69.3)	< 0.001	400 (31.8)	473 (35.5)	< 0.05
**Alcohol consumption (g/week)**							
	None	877 (43.9)	873 (30.7)	< 0.001	859 (68.2)	859 (64.5)	< 0.001
	0.1–69.9	672 (33.7)	665 (23.4)		325 (25.8)	317 (23.8)	
	70–139.9	343 (17.2)	885 (31.1)		75 (6.0)	130 (9.8)	
	140–209.9	104 (5.2)	104 (3.7)		0 (0)	9 (0.7)	
	210–279.9	0 (0)	249 (8.8)		0 (0)	10 (0.8)	
	≥ 280	0 (0)	69 (2.4)		0 (0)	7 (0.5)	
Regular exercise		563 (28.2)	838 (29.5)	0.351	191 (15.1)	198 (14.9)	0.912
Eating before going to bed		775 (38.8)	1,215 (42.7)	< 0.005	484 (38.2)	533 (40.0)	0.355
Eating breakfast		235 (11.8)	344 (12.1)	0.753	86 (6.8)	94 (7.1)	0.817
SBP	(mmHg)	129.5 ± 15.8	131.0 ± 16.0	< 0.005	129.5 ± 16.8	130.0 ± 16.8	0.460
DBP	(mmHg)	85.1 ± 11.9	86.2 ± 12.0	< 0.005	81.1 ± 11.2	81.6 ± 11.2	0.362
Hypertension		1,286 (64.4)	1,990 (69.9)	< 0.001	732 (57.7)	789 (59.2)	0.449
T-CHO	(mg/dL)	209.5 ± 35.7	211.0 ± 35.5	0.094	223.4 ± 33.6	223.7 ± 33.4	0.701
TG	(mg/dL)	151.0 ± 99.4	159.0 ± 116.9	< 0.05	120.1 ± 64.0	120.7 ± 64.0	0.776
HDL-C	(mg/dL)	53.0 ± 11.6	54.8 ± 12.6	< 0.001	64.0 ± 14.5	64.1 ± 14.7	0.891
LDL-C	(mg/dL)	134.4 ± 32.3	133.4 ± 31.9	0.429	141.3 ± 30.9	141.4 ± 29.8	0.874
Dyslipidemia		1,021 (51.2)	1,489 (52.3)	0.430	537 (42.3)	566 (42.5)	0.968
FPG	(mg/dL)	108.8 ± 24.6	108.9 ± 23.6	0.141	103.8 ± 18.7	103.8 ± 18.3	0.755
HbA1c	(%)	5.9 ± 0.90	5.9 ± 0.84	0.109	5.8 ± 0.60	5.8 ± 0.59	0.900
IGT		1,413 (70.8)	2,051 (72.1)	0.332	858 (67.7)	911 (68.4)	0.705
UA	(mg/dL)	6.2 ± 1.3	6.3 ± 1.3	0.062	5.0 ± 1.1	5.1 ± 1.1	0.395
ALT	(IU/L)	36.2 ± 22.2	35.4 ± 22.0	0.059	26.2 ± 18.1	26.3 ± 18.1	0.779
AST	(IU/L)	27.8 ± 11.3	28.5 ± 12.3	0.079	24.5 ± 9.7	24.7 ± 10.4	0.575
GGT	(IU/L)	47.9 ± 40.8	58.8 ± 62.0	< 0.001	33.3 ± 34.1	34.4 ± 36.1	0.314
AAR		0.86 ± 0.28	0.90 ± 0.31	< 0.001	1.07 ± 0.34	1.07 ± 0.34	0.933
AAR ≥ 1.0		468 (23.4)	804 (28.3)	< 0.001	637 (50.2)	673 (50.5)	0.906
APRI		0.31 ± 0.17	0.32 ± 0.22	< 0.05	0.24 ± 0.12	0.25 ± 0.14	0.591
APRI > 0.5		157 (7.9)	252 (8.9)	0.228	53 (4.2)	58 (4.4)	0.847
FIB-4 index		1.12 ± 0.49	1.19 ± 0.58	< 0.001	1.08 ± 0.41	1.09 ± 0.45	0.673
FIB-4 index ≥ 1.3 (age < 65) or ≥ 2 (age ≥ 65)		480 (24.0)	827 (29.1)	< 0.001	286 (22.6)	302 (22.7)	0.963
NFS		− 1.72 ± 1.10	− 1.62 ± 1.09	< 0.005	− 1.73 ± 1.09	− 1.70 ± 1.08	0.511
NFS ≥ − 1.455 (age < 65) or ≥ 0.12 (age ≥ 65)		804 (40.3)	1,233 (43.3)	< 0.05	502 (39.6)	539 (40.5)	0.660

Data represent the mean ± standard deviation or number (%) for categorical variables. *p*-values of two groups are based on the χ^2^-test or Mann–Whitney U-test. *p*-values of three or more groups were determined using the m × n χ^2^ test.

*AAR* AST/ALT ratio; *ALT* alanine aminotransferase; *APRI* AST-to-platelet ratio index; *AST* aspartate aminotransferase; *BMI* body mass index; *DBP* diastolic blood pressure; *FPG* fasting plasma glucose; *FIB-4* Fibrosis-4; *GGT* gamma-glutamyl transpeptidase; *HbA1c* hemoglobin A1c; *HDL-C* high-density lipoprotein cholesterol; *IGT* impaired glucose tolerance; *LDL-C* low-density lipoprotein cholesterol; *MAFLD* metabolic-associated fatty liver disease; *NAFLD* nonalcoholic fatty liver disease; *NFS* NAFLD fibrosis score; *SBP* systolic blood pressure; *T-CHO* total cholesterol; *TG* triglyceride; *UA* uric acid; *WC* waist circumference.

*p* < 0.05 was considered statistically significant.

### Comparison between NAFLD and MAFLD according to the quantity of alcohol consumption

**Figure 2:**
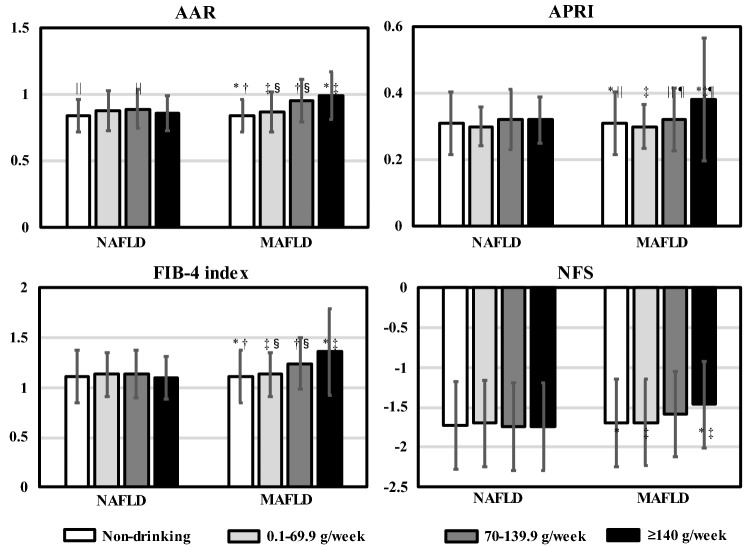
Comparison of noninvasive liver fibrosis scores between NAFLD and MAFLD according to the quantity of alcohol consumption in male patients. The white bar indicates patients without alcohol consumption. The light gray bar indicates patients with alcohol consumption of 0.1–69.9 g/week. The dark gray bar indicates patients with alcohol consumption of 70–139.9 g/week. The black bar indicates patients with alcohol consumption of ≥ 140 g/week. Data are presented as the mean ± standard deviation. * indicates a significant difference between patients without alcohol consumption and patients with alcohol consumption of ≥ 140 g/week, *p* < 0.01; † indicates a significant difference between patients without alcohol consumption and patients with alcohol consumption of 70–139.9 g/week, *p* < 0.01; ‡ indicates a significant difference between patients with alcohol consumption of 0.1–69.9 g/week and patients with alcohol consumption of ≥ 140 g/week, *p* < 0.01; § indicates a significant difference between patients with alcohol consumption of 0.1–69.9 g/week and patients with alcohol consumption of 70–139.9 g/week, *p* < 0.01; || indicates a significant difference between patients without alcohol consumption and patients with alcohol consumption of 70–139.9 g/week, *p* < 0.05; ¶ indicates a significant difference between patients with alcohol consumption of 70–139.9 g/week and patients with alcohol consumption of ≥ 140 g/week, *p* < 0.01. AAR, AST/ALT ratio; ALT, alanine aminotransferase; APRI, AST-to-platelet ratio index; AST, aspartate aminotransferase; FIB-4, Fibrosis-4; NAFLD, nonalcoholic fatty liver disease; NFS, NAFLD fibrosis score; MAFLD, metabolic-associated fatty liver disease.

In male patients, there were no significant differences in the prevalence of hypertension, dyslipidemia, and IGT; the level of liver enzymes; or noninvasive liver fibrosis scores between patients with NAFLD and those with MAFLD who were non-drinkers or consumed 0.1–69.9 g/week of alcohol (Table 5). Among male patients who consumed 70–139.9 g/week or ≥ 140 g/week of alcohol, the AAR, FIB-4 Index, and NFS were significantly higher in patients with MAFLD than in those with NAFLD (*p* < 0.005, *p* < 0.001, and* p* < 0.05, respectively). In addition, there was a significant difference in noninvasive liver fibrosis scores among the 4 groups according to the quantity of alcohol consumption in male patients with MAFLD (all *p* < 0.005) (Fig. 2). In female patients, there were no significant differences between those with NAFLD and those with MAFLD, regardless of the quantity of alcohol consumption.

**Table 5:** Comparison between NAFLD and MAFLD according to the quantity of alcohol consumption.

		Male			Female		
Alcohol consumption		NAFLD	MAFLD	*p*-value	NAFLD	MAFLD	*p*-value
(None)		(n = 877)	(n = 873)		(n = 859)	(n = 859)	
Hypertension		549 (62.6)	556 (63.7)	0.656	525 (61.1)	528 (61.5)	0.921
Dyslipidemia		453 (51.7)	459 (52.6)	0.702	381 (44.4)	383 (44.6)	0.961
IGT		648 (73.9)	659 (75.5)	0.475	597 (69.5)	604 (70.3)	0.752
ALT	(IU/L)	37.2 ± 23.6	37.2 ± 23.7	0.981	27.2 ± 19.5	27.3 ± 19.4	0.786
AST	(IU/L)	28.0 ± 12.5	28.0 ± 12.5	0.937	25.1 ± 10.4	25.1 ± 10.4	0.814
GGT	(IU/L)	43.0 ± 31.3	43.0 ± 31.3	0.990	33.9 ± 34.7	33.9 ± 34.7	0.933
AAR		0.84 ± 0.24	0.84 ± 0.25	0.925	1.05 ± 0.32	1.05 ± 0.32	0.792
APRI		0.31 ± 0.19	0.31 ± 0.19	0.996	0.25 ± 0.12	0.25 ± 0.12	0.898
FIB-4 index		1.11 ± 0.53	1.11 ± 0.53	0.929	1.08 ± 0.42	1.08 ± 0.42	0.990
NFS		− 1.72 ± 1.11	− 1.69 ± 1.10	0.606	− 1.71 ± 1.09	− 1.69 ± 1.09	0.762
Alcohol consumption		NAFLD	MAFLD	*p*-value	NAFLD	MAFLD	*p*-value
(0.1–69.9 g/week)		(n = 672)	(n = 665)		(n = 325)	(n = 317)	
Hypertension		438 (65.2)	444 (66.8)	0.564	168 (51.7)	168 (53.0)	0.752
Dyslipidemia		316 (47.0)	322 (48.4)	0.622	122 (37.5)	122 (38.5)	0.808
IGT		461 (68.6)	466 (70.1)	0.594	203 (62.5)	203 (64.0)	0.683
ALT	(IU/L)	34.4 ± 19.2	34.6 ± 19.2	0.728	23.9 ± 14.5	24.2 ± 14.6	0.748
AST	(IU/L)	26.8 ± 9.1	26.9 ± 9.0	0.833	23.3 ± 8.0	23.4 ± 8.1	0.833
GGT	(IU/L)	48.5 ± 43.9	48.9 ± 44.0	0.745	30.2 ± 27.1	30.5 ± 27.3	0.775
AAR		0.88 ± 0.31	0.87 ± 0.30	0.718	1.09 ± 0.32	1.08 ± 0.32	0.789
APRI		0.30 ± 0.12	0.30 ± 0.13	0.938	0.24 ± 0.11	0.24 ± 0.11	0.918
FIB-4 index		1.13 ± 0.45	1.13 ± 0.45	0.818	1.07 ± 0.39	1.07 ± 0.39	0.999
NFS		− 1.70 ± 1.09	− 1.69 ± 1.09	0.839	− 1.79 ± 1.05	− 1.76 ± 1.03	0.803
Alcohol consumption		NAFLD	MAFLD	*p*-value	NAFLD	MAFLD	*p*-value
(70–139.9 g/week)		(n = 343)	(n = 885)		(n = 75)	(n = 130)	
Hypertension		223 (65.0)	650 (73.4)	< 0.005	35 (46.7)	77 (59.2)	0.109
Dyslipidemia		191 (55.7)	470 (53.1)	0.444	30 (40.0)	51 (39.2)	1.000
IGT		233 (67.9)	612 (69.2)	0.681	51 (68.0)	91 (70.0)	0.756
ALT	(IU/L)	36.4 ± 23.6	33.8 ± 21.6	< 0.05	25.7 ± 15.2	26.8 ± 17.7	0.961
AST	(IU/L)	28.9 ± 12.0	28.7 ± 12.6	0.656	24.0 ± 7.4	25.9 ± 14.8	0.591
GGT	(IU/L)	54.4 ± 51.6	66.7 ± 74.7	< 0.001	41.5 ± 50.9	47.4 ± 57.4	0.088
AAR		0.89 ± 0.30	0.95 ± 0.32	< 0.005	1.14 ± 0.56	1.13 ± 0.48	0.656
APRI		0.32 ± 0.18	0.32 ± 0.19	0.726	0.23 ± 0.10	0.27 ± 0.27	0.326
FIB-4 index		1.14 ± 0.48	1.24 ± 0.51	< 0.001	1.03 ± 0.41	1.17 ± 0.73	0.170
NFS		− 1.74 ± 1.11	− 1.58 ± 1.07	< 0.05	− 1.81 ± 1.15	− 1.58 ± 1.19	0.250
Alcohol consumption		NAFLD	MAFLD	*p*-value	NAFLD	MAFLD	*p*-value
(≥ 140 g/week)		(n = 104)^†^	(n = 422)		(n = 0)	(n = 26)	
Hypertension		76 (73.1)	340 (80.6)	0.092	(–)	16 (61.5)	NA
Dyslipidemia		61 (58.7)	238 (56.4)	0.677	(–)	10 (38.5)	NA
IGT		71 (68.3)	314 (74.4)	0.267	(–)	13 (50.0)	NA
ALT	(IU/L)	38.9 ± 22.2	36.1 ± 23.1	0.074	(–)	20.0 ± 7.7	NA
AST	(IU/L)	29.7 ± 10.9	31.5 ± 14.6	0.532	(–)	23.7 ± 5.5	NA
GGT	(IU/L)	64.7 ± 43.3	90.4 ± 85.9	< 0.005	(–)	33.5 ± 25.3	NA
AAR		0.86 ± 0.27	0.99 ± 0.36	< 0.005	(–)	1.25 ± 0.28	NA
APRI		0.32 ± 0.14	0.38 ± 0.37	0.261	(–)	0.25 ± 0.77	NA
FIB-4 index		1.10 ± 0.43	1.36 ± 0.87	< 0.001	(–)	1.17 ± 0.44	NA
NFS		− 1.74 ± 1.09	− 1.46 ± 1.09	< 0.05	(–)	− 1.71 ± 1.11	NA

Data are presented as the mean ± standard deviation or number (%) for categorical variables. *p*-values are based on the χ^2^-test or Mann–Whitney U-test.

*AAR* AST/ALT ratio; *ALT* alanine aminotransferase; *APRI* AST-to-platelet ratio index; *AST* aspartate aminotransferase; *FIB-4* Fibrosis-4; *GGT* gamma-glutamyl transpeptidase; *IGT* impaired glucose tolerance; *MAFLD* metabolic-associated fatty liver disease; *NA* not applicable; *NAFLD* nonalcoholic fatty liver disease; *NFS* NAFLD fibrosis score.

*p* < 0.05 was considered statistically significant.

^†^NAFLD participants with alcohol consumption of 140–209.9 g/week.

### Comparison of qualitative abdominal fat between NAFLD and MAFLD patients

At baseline, there were no significant differences in age, sex, body mass index (BMI), or waist circumference (WC) between all patients with NAFLD (n = 3,264) and those with NAFLD who underwent measurement of abdominal fat by CT (n = 1,340, 41.1%) or between all the patients with MAFLD (n = 4,177) and those with MAFLD who underwent measurement of abdominal fat by computed tomography (CT) (n = 1,807, 43.3%). There were no significant differences in BMI, WC, total adipose area (TAA), or subcutaneous adipose area (SAA) between patients with NAFLD and those with MAFLD in either sex (Table 6). In male patients, visceral adipose area (VAA), VAA-to-SAA ratio (VAA/SAA), and the prevalence of VAA ≥ 100 cm^2^ and VAA/SAA ≥ 1 were significantly higher in patients with MAFLD than in those with NAFLD (*p* < 0.001,* p* < 0.05,* p* < 0.001, and* p* < 0.05, respectively). In female patients, VAA and the prevalence of VAA ≥ 100 cm^2^ were significantly higher in patients with MAFLD than in those with NAFLD (both *p* < 0.001).

**Table 6:** Comparison of qualitative abdominal fat between NAFLD and MAFLD.

		Male			Female		
		NAFLD	MAFLD	*p*-value	NAFLD	MAFLD	*p*-value
Number		1,014	1,465		326	342	
BMI	(kg/m^2^)	26.6 ± 3.4	26.4 ± 3.2	0.285	26.2 ± 4.1	26.4 ± 4.1	0.468
WC	(cm)	91.5 ± 8.7	91.4 ± 8.4	0.977	89.8 ± 9.2	90.4 ± 9.1	0.413
TAA	(cm^2^)	320.3 ± 98.8	318.9 ± 96.2	0.845	351.1 ± 106.1	355.9 ± 105.3	0.512
SAA	(cm^2^)	193.3 ± 74.2	189.8 ± 72.0	0.240	246.2 ± 87.4	249.8 ± 87.1	0.516
VAA	(cm^2^)	108.1 ± 34.1	140.7 ± 46.0	< 0.001	96.4 ± 27.8	120.8 ± 42.7	< 0.001
VAA (≥ 100 cm^2^)		606 (59.8)	1,192 (81.4)	< 0.001	141 (43.4)	211 (61.7)	< 0.001
VAA/SAA		0.72 ± 0.30	0.75 ± 0.31	< 0.05	0.46 ± 0.18	0.45 ± 0.19	0.884
VAA/SAA (≥ 1)		146 (14.4)	256 (17.5)	< 0.05	7 (2.1)	8 (2.3)	1.000

Data are presented as the mean ± standard deviation or number (%) for categorical variables. *p*-values are based on the χ^2^-test or Mann–Whitney U-test.

*BMI* body mass index; *MAFLD* metabolic-associated fatty liver disease; *NAFLD* nonalcoholic fatty liver disease; *SAA* subcutaneous adipose area; *TAA* total adipose area; *VAA* visceral adipose area; *VAA/SAA* VAA-to-SAA ratio; *WC* waist circumference.

*p* < 0.05 was considered statistically significant.

## Discussion

The present study highlights the differences in clinical factors within the MAFLD group based on the number of MAFLD components. The principal findings were that noninvasive liver fibrosis scores and qualitative evaluation of abdominal fat were useful for distinguishing between NAFLD and MAFLD in male patients. In addition, although there was very little difference between NAFLD and MAFLD in female patients, regardless of the quantity of alcohol consumption, several noninvasive liver fibrosis scores were significantly higher in patients with MAFLD than in those with NAFLD among males who consumed > 70 g/week of alcohol.

The level of liver enzymes (including AST and ALT) and the prevalence of metabolic dysregulations (such as hypertension, dyslipidemia, and IGT) increased significantly with an increase in the number of MAFLD components. In addition, these factors were associated with the onset of MAFLD in the present study, which was in accordance with findings of previous reports on NAFLD^15,16^. Regular exercise has been shown to reduce the risk of NAFLD^17^; the present study showed that regular exercise reduced the risk of MAFLD in both the sexes. Recent studies reported that considering metabolic condition rather than obese on metabolic fatty liver was important because not a few non-obese individuals existed in metabolic fatty liver population^18,19^. In the present study, among patients with and without fatty liver excepting obese patients, the prevalence of fullness for MAFLD criteria except diagnosis of ultrasonography was 88.4% and 58.6%, respectively (*p* < 0.001). Our results supported the importance of considering metabolic abnormality on the development of MAFLD.

Regarding noninvasive liver fibrosis scores, the present study showed that increases in APRI and NFS and decreases in AAR and FIB-4 Index were correlated with MAFLD. We hypothesized that the AAR is not suitable for assessing liver fibrosis in participants with non-MAFLD or mild MAFLD because in non-MAFLD patients without fatty liver, AAR was > 1 in 86.5% (5,071/5,865) of participants with normal ALT (< 30 IU/L in male patients and < 19 IU/L in female patients) and 49.3% (816/1,654) of patients with elevated ALT levels. Additionally, age < 35 years and age > 65 years has been reported to be a potential confounding factor for the FIB-4 Index and AAR^20,21^. Further clinical studies on noninvasive liver fibrosis scores for MAFLD investigating different age groups and liver fibrosis are required.

Obesity is generally categorized based on the location of adipose accumulation as subcutaneous and visceral. The latter is considered to markedly contribute to the development of various digestive diseases, including NAFLD^22–34^. However, the influence of qualitative abdominal fat on MAFLD is unclear. The present study showed that VAA/SAA was significantly higher in patients with MAFLD than in those with NAFLD in male patients only. These results may reflect sex differences based on adipose tissue and hormones; regional fat distribution is known to be associated with the risk of metabolic disorders and NAFLD, with a lower risk resulting from gynoid gluteo-femoral subcutaneous distribution and a higher risk with android visceral adiposity^26,27^. Adiponectin and estradiol, which are higher in female individuals, reduce lipolysis and improve adipose tissue insulin sensitivity^28–30^. In addition, there were no significant differences in BMI, WC, TAA, or SAA between patients with NAFLD and those with MAFLD; many factors associated with metabolic dysregulation were higher in patients with MAFLD than in those with NAFLD, suggesting that WC may be a marker of visceral fat. However, WC cannot reflect the ratio of visceral fat and subcutaneous fat and visceral fat may be more strongly associated with MAFLD than NAFLD.

Alcohol consumption is known to be an essential factor for advanced liver fibrosis in patients with MAFLD; therefore, the concept of MAFLD was established for the early detection of advanced fibrosis^8^. The influence of alcohol intake is known to differ between sexes^31–33^; alcohol-related liver disease is more common in male patients because males consume more alcohol than females and females are more easily affected by alcohol than males^34,35^. The present study showed that the prevalence of drinking in male patients was significantly higher than that in female patients among all participants (70.9% vs. 40.2%; Table 1), patients with NAFLD (56.1% vs. 31.8%; Table 4), and patients with MAFLD (69.3% vs. 35.5%; Table 4). Although alcohol consumption contributed to the decrease of MAFLD in male participants regardless of the quantity, alcohol consumption of > 280 g/week might contribute to the increase of MAFLD in female participants. Among male patients with an alcohol consumption of > 70 g/week, several noninvasive liver fibrosis scores were significantly higher in patients with MAFLD than in those with NAFLD. These results suggest that the influence of alcohol consumption in female patients may be small compared to the influence on the discrepancy between NAFLD and MAFLD in male patients, and male MAFLD patients with an alcohol consumption of > 70 g/week may be prone to developing liver fibrosis.

The strength of the present study is the use of ultrasonography, which is simple, noninvasive, widely used, and accurate in the evaluation of steatosis. The sensitivity and specificity of ultrasonography for the detection of ≥ 5% and ≥ 30% of steatotic hepatocytes on histology were reported as 82%, 80% and 85%, 85%, respectively, in a recent meta-analysis^36^. Additionally, no reports about usefulness of noninvasive liver fibrosis scores and qualitative evaluation of abdominal fat for diagnosis of distinguishing between NAFLD and MAFLD. These facts make the results convincing. However, several limitations exist in the present study that should be acknowledged. First, it was a single-center observational study. Therefore, multi-center studies are needed to validate our findings. Second, there was a possibility of selection bias because the most participants were voluntary attendees who underwent a self-paid medical check-up and were restricted to office workers of middle and high socioeconomic status. Additionally, whether patients hospitalized for MAFLD or NAFLD would yield similar results remains unclear. Further large-scale clinical investigations on the differences between these groups are needed. Third, only 43% of patients with MAFLD underwent CT scanning to measure abdominal fat. CT is not always performed during medical check-ups because the necessary equipment is only available in relatively large-scale medical institutions and it is mildly associated with radiation exposure. Finally, we did not obtain detailed information regarding medications for hypertension, dyslipidemia, and diabetes mellitus and diets including volume, calories, and contents.

In conclusion, noninvasive liver fibrosis scores and qualitative evaluation of abdominal fat were useful for distinguishing between NAFLD and MAFLD in male patients. The influence of alcohol consumption on the discrepancy between NAFLD and MAFLD was different between male and female participants, and the development of liver fibrosis should be considered in male patients with MAFLD who exceed mild drinking.

## Methods

### Study design and participants

This cross-sectional study included 12,985 adults undergoing regular health check-ups at Shikoku Central Hospital of the Mutual Aid Association of Public School Teachers between April 2016 and March 2018. After excluding participants who had incomplete information, underwent prior liver surgery, visited the hospital for treatment, or were followed up for liver diseases such as alcoholic, viral, and drug-induced liver disease, 11,766 patients were finally analyzed (Figure S1). The study design was approved by the Ethics Committees of Shikoku Central Hospital of the Mutual Aid Association of Public School Teachers, and the study was performed in conformance with the Declaration of Helsinki. Regarding patient consent, an opt-out approach was used in this study, and personal information was protected during data collection.

### Clinical assessment

Drug history, hospital admission data, and lifestyle habits were recorded using a standardized questionnaire, and health check-up nurses interviewed participants individually to confirm each item on the questionnaire. In the present study, current smoking excluded previous smoking. The amount of alcohol consumed per drinking day was determined in grams using representative percent alcohol by volume for each type of alcohol: 5% for beer, 16% for Japanese sake, 25% for shochu, 10% for wine, and 34% for whiskey. Based on the drinking information, patients were divided into two categories: non-drinkers [participants drinking 12 drinks or less per year of < 20 g/drinking day] and drinkers [participants whose drinking exceeded the abovementioned measurements]. Excessive alcohol consumption was defined as > 30 g and > 20 g of daily alcohol consumption for males and females, respectively^37,38^. The average weekly alcohol consumption was classified into five categories: none, 0.1–69.9 g/week, 70–139.9 g/week, 140–279.9 g/week, and ≥ 280 g/week.

Regular exercise was defined as performing a > 30-min exercise session at least once per week. The habit of eating before going to bed was defined as eating within 2 h before going to bed at least once per week. When participants underwent regular health check-ups with abdominal fat on CT, one slice was acquired at the level of the navel to measure the VAA and SAA^39^, which are indices of the metabolic syndrome^40^.

Venous blood samples were obtained from all the participants in the morning after 12 h of overnight fasting. The following clinical laboratory parameters were evaluated: AST, ALT, GGT, total cholesterol (T-CHO), high-density lipoprotein cholesterol (HDL-C), triglyceride (TG), low-density lipoprotein cholesterol (LDL-C), uric acid (UA), fasting plasma glucose (FPG), and hemoglobin A1c (HbA1c). The AAR, APRI, FIB-4 Index, and NFS were calculated to evaluate the liver, referring to published formulas and cut-offs^41^.

Hypertension was defined as blood pressure (BP) ≥ 130/85 mmHg or the use of medications for hypertension. Dyslipidemia was defined as a TG level ≥ 150 mg/dL, HDL-C level < 40 mg/dL for males and < 50 mg/dL for females, or the use of medications for dyslipidemia. IGT was defined as FPG level ≥ 100 mg/dL or the use of medications for diabetes mellitus.

### Diagnostic criteria for NAFLD and MAFLD

NAFLD was defined by the evidence of hepatic steatosis on ultrasound and the exclusion of excessive alcohol consumption and other competing causes for hepatic steatosis (e.g., viral hepatitis)^37,38^. The criteria for hepatic steatosis on ultrasonography were as follows: increased hepatorenal echo contrast, liver brightness, vessel blurring, and/or deep attenuation^42^. MAFLD was defined by the evidence of hepatic steatosis on ultrasound and the presence of any of the following criteria: overweight/obesity, presence of type 2 diabetes mellitus (T2DM), and evidence of metabolic dysregulation^8,9^.

Overweight was defined as a BMI of ≥ 23 kg/m^2^ in Asians. The presence of metabolic dysregulation was defined as the presence of two or more of the following metabolic conditions: WC ≥ 90 cm in male patients and ≥ 80 cm in female patients; BP ≥ 130/85 mmHg or specific drug treatment; TG level ≥ 150 mg/dL or specific drug treatment; HDL-C level < 40 mg/dL in male patients and < 50 mg/dL in female patients or specific drug treatment; and prediabetes (FPG level of 100–125 mg/dL or HbA1c level of 5.7–6.4%). Although the high-sensitivity C-reactive protein (CRP) level and the homeostatic model assessment for insulin resistance (HOMA-IR) score reflect metabolic dysregulation, these assessments are not generally conducted in Japanese medical check-ups. Therefore, high-sensitivity CRP and HOMA-IR measurements were not available in the present study.

Because it is unclear whether MAFLD severity is reflected in clinical practice, patients who fulfilled the MAFLD criteria were classified into three groups according to the number of the abovementioned MAFLD components that were fulfilled (overweight/obesity, T2DM, and metabolic dysregulation). Therefore, Group 1, Group 2, and Group 3 indicated having one, two, and three MAFLD components, respectively.

### Statistical analysis

Continuous variables are presented as the mean ± standard deviation, and categorical data are presented as counts (percentages). Differences were considered to be statistically significant at *p* < 0.05. Comparisons of the proportions and categorical variables between two groups and two additional groups were performed using the χ^2^ test and the m × n χ^2^ test, respectively. According to the data, the distribution was not normal, so the Mann–Whitney U and Kruskal–Wallis nonparametric tests were used between two groups and two additional groups, respectively. If the Kruskal–Wallis test revealed differences between the groups, post-hoc pairwise comparisons were performed using the Mann–Whitney U-test with Bonferroni correction. Factors with significant influence on the prevalence of MAFLD were determined using multivariate logistic regression analysis including adjustments for age, BMI, and WC. The OR and 95% CI were analyzed for each variable. All statistical analyses were performed using MedCalc Statistical Software for Windows (MedCalc Software, Ostend, Belgium).

### Ethical statement

The study design was approved by the Ethics Committees of Shikoku Central Hospital of the Mutual Aid Association of Public School Teachers, and the study was performed in conformance with the Declaration of Helsinki. Regarding patient consent, an opt-out approach was used in this study, and personal information was protected during data collection.

## Supplementary Information

Supplementary Information.

## Data Availability

All data generated or analyzed during this study are included in this published article.
