# Supplementary material for: Comparison of the role of alcohol consumption and qualitative abdominal fat on NAFLD and MAFLD in males and females
Source: Sci Rep. 2022 Sep 26;12:16048. doi: 10.1038/s41598-022-20124-8 (PMC9512786; doi:10.1038/s41598-022-20124-8)
Supplement: Supplementary file 1 — Supplementary Information. [file 41598_2022_20124_MOESM1_ESM.pdf]

**Supplementary Information**

**Supplementary Table S1** Comparison of characteristics between NAFLD and MAFLD in female patients according to age

| | | Female (age < 50 years) | | | Female (age ≥ 50 years) | | |
|---|---|---|---|---|---|---|---|
| | | NAFLD | MAFLD | *p*-value | NAFLD | MAFLD | *p*-value |
| Number | | 296 | 302 | | 973 | 1,030 | |
| BMI | ($kg/m^2$) | 28.1 ± 4.9 | 28.3 ± 4.8 | 0.459 | 26.2 ± 3.7 | 26.2 ± 3.7 | 0.714 |
| WC | (cm) | 92.4 ± 10.8 | 93.1 ± 10.3 | 0.369 | 90.2 ± 8.9 | 90.5 ± 8.9 | 0.482 |
| Current smoking | | 1 (0.3) | 1 (0.3) | 0.969 | 11 (1.1) | 15 (1.5) | 0.520 |
| Drinking | | 104 (36.4) | 121 (40.1) | 0.356 | 296 (30.4) | 352 (34.2) | 0.073 |
| Alcohol consumption (g/week) | | | | | | | |

| | | | | | | | |
|---|---|---|---|---|---|---|---|
| None | | 182 (63.6) | 181 (59.9) | < 0.05 | 677 (69.6) | 678 (65.8) | < 0.001 |
| 0.1–69.9 | | 83 (29.0) | 79 (26.2) | | 242 (24.9) | 238 (23.1) | |
| 70–139.9 | | 21 (7.3) | 30 (9.9) | | 54 (5.5) | 100 (9.7) | |
| 140–279.9 | | 0 (0) | 7 (2.3) | | 0 (0) | 12 (1.2) | |
| ≥ 280 | | 0 (0) | 5 (1.7) | | 0 (0) | 2 (0.2) | |
| Regular exercise | | 22 (7.7) | 23 (7.6) | 0.972 | 167 (17.2) | 175 (17.0) | 0.918 |
| Eating before going to bed | | 118 (41.3) | 133 (44.0) | 0.496 | 360 (37.0) | 400 (38.8) | 0.397 |
| Eating breakfast | | 25 (8.7) | 26 (8.6) | 0.955 | 60 (6.2) | 68 (6.6) | 0.690 |
| SBP | (mmHg) | 126.6 ± 17.1 | 126.9 ± 16.9 | 0.811 | 130.4 ± 16.6 | 130.9 ± 16.7 | 0.466 |
| DBP | (mmHg) | 81.1 ± 12.5 | 81.5 ± 12.2 | 0.763 | 81.1 ± 10.8 | 81.6 ± 10.9 | 0.393 |
| Hypertension | | 134 (46.9) | 143 (47.4) | 0.904 | 594 (61.0) | 646 (62.7) | 0.442 |

| | | | | | | | |
|---|---|---|---|---|---|---|---|
| T-CHO | (mg/dL) | 213.0 ± 32.7 | 214.1 ± 31.9 | 0.624 | 226.5 ± 33.3 | 226.6 ± 33.3 | 0.883 |
| TG | (mg/dL) | 120.1 ± 62.6 | 122.4 ± 62.6 | 0.566 | 120.1 ± 64.4 | 120.1 ± 64.5 | 0.981 |
| HDL-C | (mg/dL) | 58.7 ± 13.2 | 58.4 ± 12.9 | 0.772 | 65.5 ± 14.5 | 65.8 ± 14.8 | 0.752 |
| LDL-C | (mg/dL) | 137.0 ± 28.5 | 138.2 ± 28.2 | 0.623 | 142.5 ± 30.4 | 142.4 ± 30.2 | 0.906 |
| Dyslipidemia | | 114 (39.9) | 122 (40.4) | 0.894 | 419 (43.1) | 444 (43.1) | 0.984 |
| FPG | (mg/dL) | 102.5 ± 22.1 | 102.3 ± 21.5 | 0.996 | 104.2 ± 17.6 | 104.3 ± 17.3 | 0.791 |
| HbA1c | (%) | 5.8 ± 0.74 | 5.8 ± 0.72 | 0.937 | 5.9 ± 0.55 | 5.8 ± 0.54 | 0.835 |
| IGT | | 156 (54.5) | 167 (55.3) | 0.855 | 695 (71.4) | 744 (72.2) | 0.689 |
| UA | (mg/dL) | 4.9 ± 1.0 | 5.0 ± 1.0 | 0.534 | 5.1 ± 1.1 | 5.1 ± 1.1 | 0.478 |
| ALT | (IU/L) | 27.5 ± 21.2 | 27.9 ± 21.0 | 0.671 | 25.9 ± 17.1 | 25.9 ± 17.1 | 0.981 |
| AST | (IU/L) | 24.0 ± 11.9 | 24.2± 11.6 | 0.630 | 24.7 ± 9.0 | 24.9 ± 9.9 | 0.677 |

| | | | | | | | |
|---|---|---|---|---|---|---|---|
| GGT | (IU/L) | 29.9 ± 23.4 | 30.3 ± 23.3 | 0.678 | 34.4 ± 36.7 | 35.6 ± 39.0 | 0.379 |
| AAR | | 1.03 ± 0.37 | 1.02 ± 0.39 | 0.740 | 1.08 ± 0.33 | 1.08 ± 0.33 | 0.687 |
| AAR ≥ 1.0 | | 126 (44.1) | 131 (43.4) | 0.868 | 504 (51.8) | 542 (52.6) | 0.713 |
| APRI | | 0.21 ± 0.12 | 0.22 ± 0.12 | 0.564 | 0.25 ± 0.11 | 0.26 ± 0.14 | 0.711 |
| APRI > 0.5 | | 16 (5.6) | 16 (5.3) | 0.874 | 37 (3.8) | 42 (4.1) | 0.752 |
| FIB-4 index | | 0.75 ± 0.27 | 0.75 ± 0.26 | 0.667 | 1.18 ± 0.39 | 1.19 ± 0.45 | 0.650 |
| FIB-4 index ≥ 1.3 (age < 65 years) or ≥ 2 (age ≥ 65 years) | | | | | | | |
| | | 14 (4.9) | 13 (4.3) | 0.732 | 268 (27.5) | 289 (28.1) | 0.797 |
| NFS | | -2.50 ± 1.05 | -2.44 ± 1.04 | 0.496 | -1.51 ± 0.99 | -1.48 ± 1.00 | 0.558 |
| NFS ≥ -1.455 (age < 65 years) or ≥ 0.12 (age ≥ 65 years) | | | | | | | |
| | | 48 (16.8) | 54 (17.9) | 0.725 | 449 (46.2) | 485 (47.1) | 0.674 |

**Notes:** Data represent the mean ± standard deviation or number (%) for categorical variables. *p*-values of two groups are based on the $\chi^2$-test

or Mann–Whitney U-test. $p$-values of three or more groups were determined using the m × n $\chi^2$ test. $p < 0.05$ was considered statistically significant.

AAR, AST/ALT ratio; ALT, alanine aminotransferase; APRI, AST-to-platelet ratio index; AST, aspartate aminotransferase; BMI, body mass index; DBP, diastolic blood pressure; FPG, fasting plasma glucose; FIB-4, Fibrosis-4; GGT, gamma-glutamyl transpeptidase; HbA1c, hemoglobin A1c; HDL-C, high-density lipoprotein cholesterol; IGT, impaired glucose tolerance; LDL-C, low-density lipoprotein cholesterol; MAFLD, metabolic-associated fatty liver disease; NAFLD, non-alcoholic fatty liver disease; NFS, NAFLD fibrosis score; SBP, systolic blood pressure; T-CHO, total cholesterol; TG, triglyceride; UA, uric acid; WC, waist circumference.

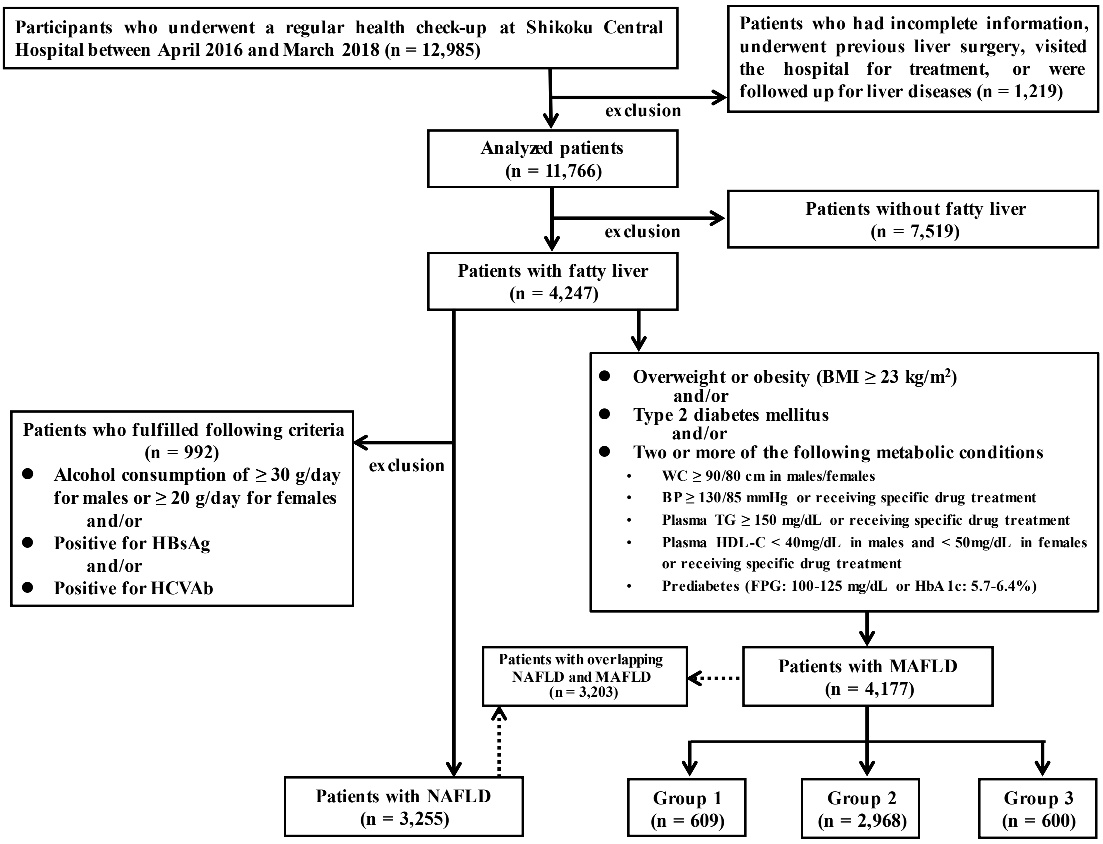


**Supplementary Figure S1** Participant flow diagram of individuals undergoing health check-ups.

BMI, body mass index; BP, blood pressure; FPG, fasting plasma glucose; HbA1c, hemoglobin A1c; HBsAg, hepatitis B surface antigen; HCV Ab, hepatitis C antibody; HDL-C, high-density lipoprotein cholesterol; MAFLD, metabolic-associated fatty liver disease; NAFLD, nonalcoholic fatty liver disease; TG; triglyceride; WC; waist circumference.
